# Lateral Deviation of the Tongue in an Otherwise Asymptomatic Patient

**DOI:** 10.1111/odi.70063

**Published:** 2025-10-22

**Authors:** Giulia Ghidini, Alessandro Olivari, Sebastiano Buti, Paolo Vescovi, Maddalena Manfredi

**Affiliations:** ^1^ Oral Surgery Resident Università Degli Studi di Milano Statale–ASST Santi Carlo e Paolo Milan Italy; ^2^ Centro di Odontoiatria, Dipartimento di Medicina e Chirurgia Università Degli Studi di Parma Parma Italy; ^3^ Unità di Odontostomatologia, Dipartimento Testa e Collo Azienda Ospedaliero‐Universitaria di Parma Parma Italy; ^4^ Medical Oncology Unit University Hospital of Parma Parma Italy

**Keywords:** laterodeviation, lung adenocarcinoma, oral metastasis, tongue, tongue lateral deviation

## 1 Case Report

**FIGURE 1:**
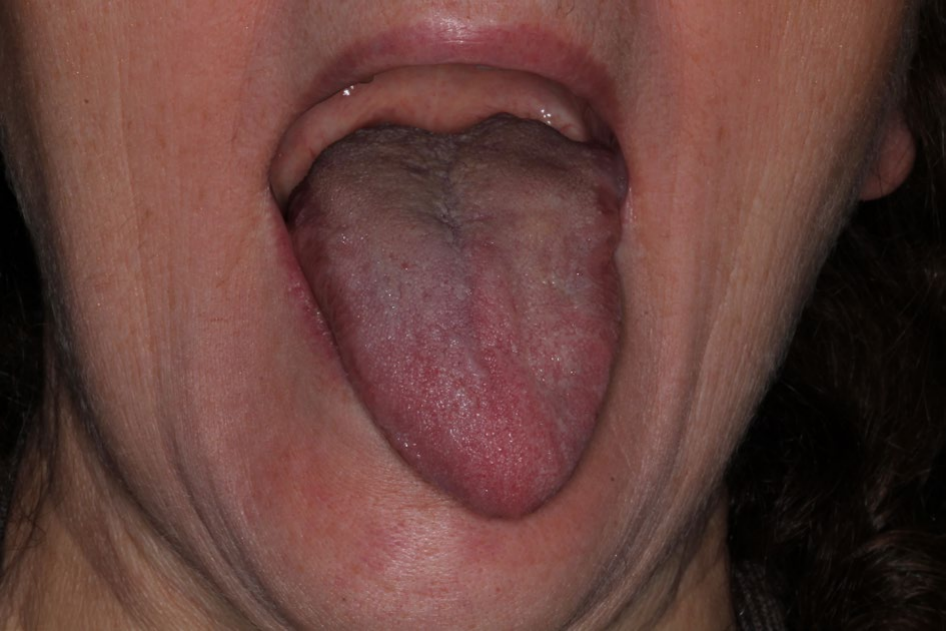
Patient's tongue during first visit.

A 61‐year‐old female patient came to our emergency department in February 2016 complaining of a recently onset feeling of enlargement at the base of the tongue, associated with mild difficulty in swallowing. She did not report any tongue mobility impairment nor speech difficulties. The patient's medical record was negative for drug's intake; the patient reported a mild mitral valve insufficiency, monitored by the cardiologist, and no prior heart surgery. At the intraoral examination, a swelling of the left posterior tongue was observed, particularly during tongue protrusion (Figure 1A,B), whereas tongue sensitivity and taste perception were preserved. No other lesions were observed in the oral cavity as well as in the head and neck area.

Based on the patient's history, physical examination, and laboratory findings, which one of the following is the most suspicious diagnosis?

(A) Neurinoma of the 12th cranial nerve.

(B) Stroke.

(C) Metastasis.

(D) Carotid dissection.

Correct answer: C.

The patient reported a sensation of swelling at the base of the tongue, which upon inspection did not correspond to an oral lesion. However, during tongue protrusion or certain lateral movements, a visible swelling became apparent. This type of clinical presentation was attributable to a nerve compression of central origin, which prompted the request for an magnetic resonance imaging (MRI). The imaging revealed the presence of multiple metastatic lesions.

**FIGURE 2:**
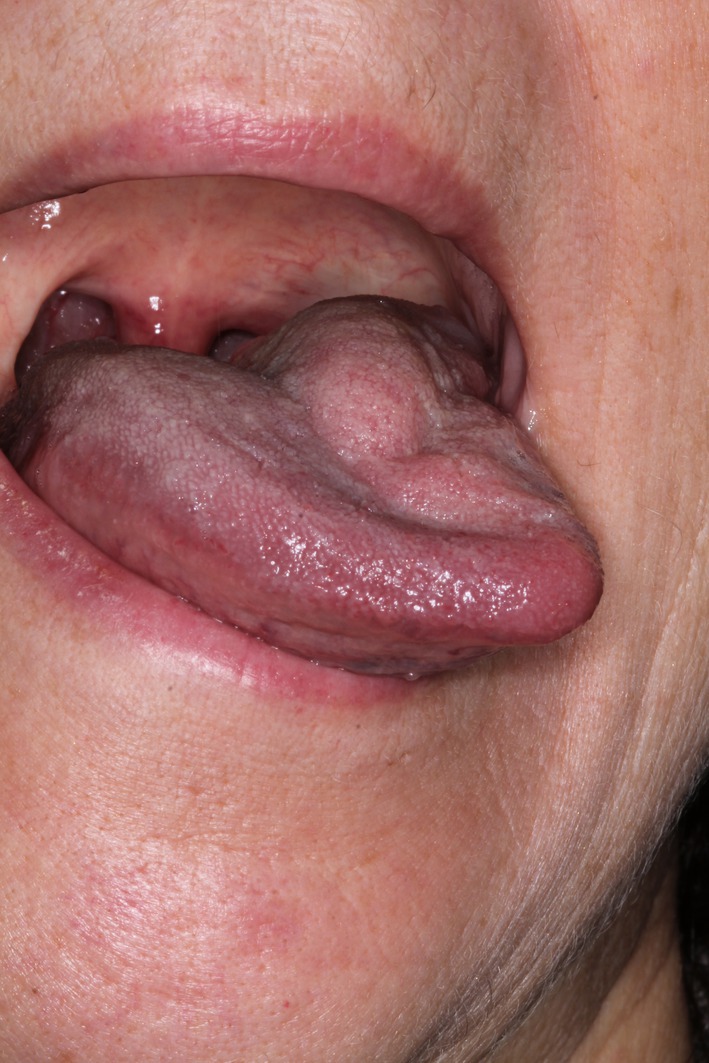
Patiet's tongue mobility impairment during first visit.

**FIGURE 3:**
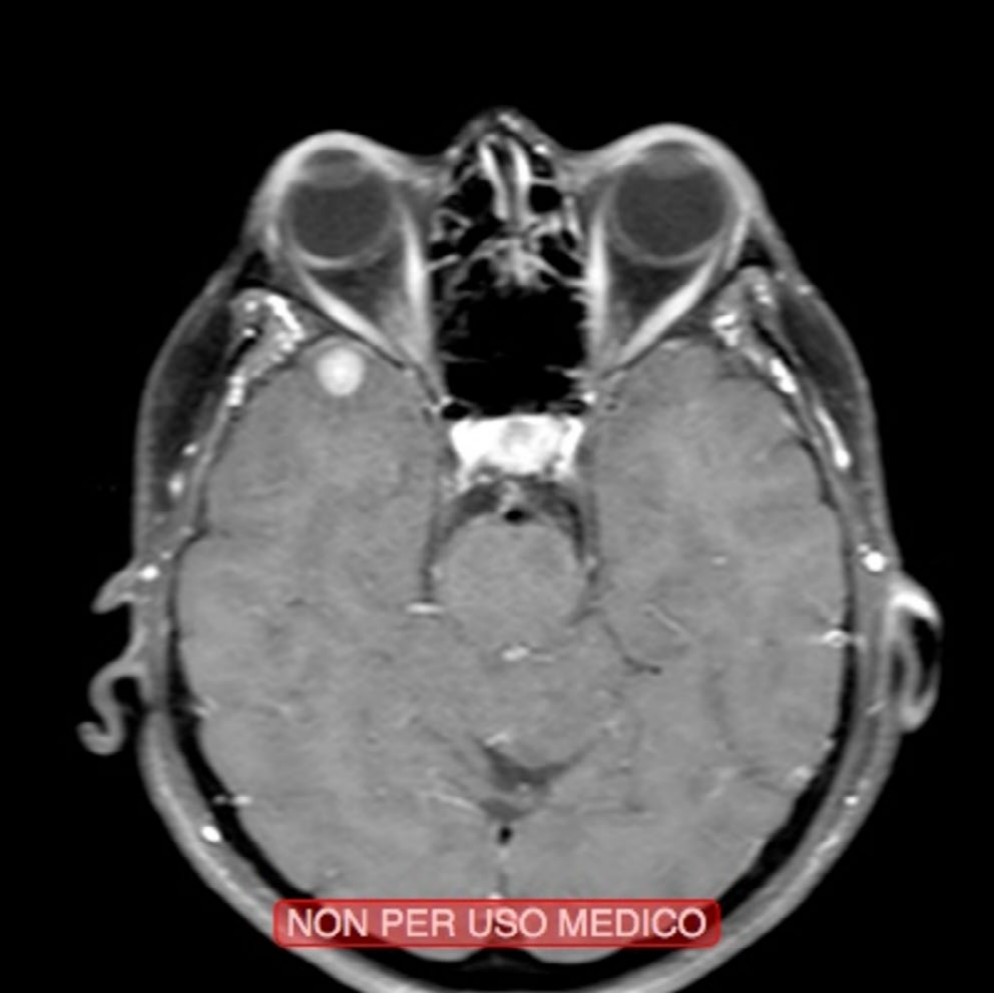
Transversal MRI section showing intracranial lesions, later identified as lung adenocarcinoma metastasis.

A head and neck contrast‐enhanced MRI was performed the following week (Figure 2). Brain MRI showed four intracranial lesions localized in different areas; these lesions were regarded as localizations of tumor of unknown primary origin. A total body computerized axial tomography scan (CT scan) with contrast medium was subsequently performed. The CT scan highlighted a noncalcified solid nodule in the right upper lung lobe with a major diameter measuring 2.5 cm. Moreover, several osteolytic lesions located in ribs, vertebral (D9‐L4), and in the right ileo‐pubic area were also highlighted, compatible with secondary lesions (Figure 3).

A diagnosis of stage IV lung adenocarcinoma with bone and brain involvement was made on a biopsy of a rib lesion in March 2016. The tumor was found to be characterized by exon 21 (L858R) epidermal growth factor receptor (EGFR) mutation.

## 2 Discussion

Metastases from malignant tumors in the oral cavity are rare and represent approximately 1% of all oral neoplasms, being more frequent in the jaw than in the soft tissues (Amro et al. 2014). The most common oral sites are the gingiva, the alveolar mucosa, and the tongue (Capodiferro et al. 2021).

The oral cavity has been shown to be a site of possible metastasis of primary solid tumors: such as breast and genital organs for women, whereas lung, kidney, and skin for men (Hirshberg et al. 1993).

Even if it is reported in the literature, the oral region is an uncommon site for metastatic tumor cell colonization, and it is usually a manifestation of a widespread disease. Frequently, oral metastases were found to be the first sign of a wide metastatic tumor spread of an undiscovered malignancy at a distant site. (Hirshberg et al. 2008).

Lung cancers mostly metastasize to the bones, liver, lymph nodes, brain, lung, and adrenal glands, with adenocarcinoma being the most common histological type. Lung metastases to the oral region are very uncommon (Gargouri et al. 2018) and manifest as gingival exophytic masses, as reported by Donís et al. (2021) in a systematic review conducted in 2021.

Disorders of the 12th cranial nerve can cause weakness or atrophy of the tongue on the affected side, as this nerve is the main responsible for tongue movements.

Patients with a hypoglossal nerve disorder usually present with speaking, chewing, and swallowing difficulty or impairment.

Even if tumors are the most frequent cause of hypoglossal nerve deficit, stroke, or brain stem infection must also be taken into consideration during the process of differential diagnosis.

Moreover, neck injuries, due to surgical removal of an obstruction of an artery in the neck (i.e., endarterectomy) or amyotrophic lateral sclerosis (also known as Lou Gehrig's disease) could also lead to similar symptoms to those described in this case report. Furthermore, hypoglossal nerve palsy secondary to carotid dissection, although it is a rare eventuality, must also be taken into account in differential diagnosis as it has already been described in the literature (Lombardi et al. 2021).

Because of its rarity, the diagnosis and primary tumor site localization of a metastatic lesion in the oral region are challenging, both to the clinician and to the pathologist.

This case report also emphasizes the need to evaluate and consider treatment options with the aim to relieve complications in the mouth, even if the prognosis of the primary tumors remains poor.

## 3 Outcome

In April 2016, Afatinib, a second‐generation specific oral tyrosine‐kinase inhibitor, was started at a dose of 40 mg per day.

The patient underwent stereotactic radiotherapy for the occipital bone lesion.

Due to gastrointestinal (diarrhea) and skin toxicity, treatment with Afatinib was switched to Osimertinib at a dose of 80 mg per day in March 2019, with a clear partial response to target therapy. In December 2017, disease progression occurred only in the brain, treated with several stereotactic RT sessions between December 2017 and December 2020. Due to meningeal disease progression occurring in May 2022, our patient was started on Osimertinib (80 mg per day) plus chemotherapy with Cisplatin and Pemetrexed, obtaining clinical benefit. The patient is still on treatment with Pemetrexed maintenance and Osimertinib.

After 6 years of closely monitored follow‐up, the tongue disease remains stable and the patient is in good clinical condition.

## Author Contributions

**Ghidini Giulia:** conceptualization, writing – original draft, writing – review and editing, data curation, investigation. **Alessandro Olivari:** investigation, formal analysis. **Sebastiano Buti:** investigation, formal analysis, supervision, methodology. **Paolo Vescovi:** investigation, methodology. **Manfredi Maddalena:** conceptualization, investigation, writing – original draft, writing – review and editing, validation, supervision, methodology.

## Conflicts of Interest

The authors declare no conflicts of interest.

## Data Availability

Data sharing not applicable to this article as no datasets were generated or analysed during the current study.

## References

[odi70063-bib-0001] Amro, L. , L. Maliki , B. Belabidia , and A. Yazidi . 2014. “Gingival Metastasis Revealing Lung Adenocarcinoma.” ALC 3: 35–37.

[odi70063-bib-0002] Capodiferro, S. , L. Limongelli , and G. Favia . 2021. “Oral and Maxillo‐Facial Manifestations of Systemic Diseases: An Overview.” Medicina 57, no. 3: 271. 10.3390/medicina57030271. 33809659 PMC8002330

[odi70063-bib-0003] Donís, S. P. , A. G. García , P. G. Vila , et al. 2021. “Gingival Exophytic Lesions as First Oral Manifestation of Primary Lung Adenocarcinomas: Systematic Review.” National Journal of Maxillofacial Surgery 12, no. 3: 297–302. 10.4103/njms.NJMS_120_20. 35153422 PMC8820301

[odi70063-bib-0004] Gargouri, I. , W. H. Benzarti , S. Aissa , et al. 2018. “Lung Adenocarcinoma With Gingival Metastasis.” European Journal of Case Reports in Internal Medicine 5, no. 6: 000861. 10.12890/2018_000861. 30756039 PMC6346877

[odi70063-bib-0005] Hirshberg, A. , P. Leibovich , and A. Buchner . 1993. “Metastases to the Oral Mucosa: Analysis of 157 Cases.” Journal of Oral Pathology & Medicine 22, no. 9: 385–390. 10.1111/j.1600-0714.1993.tb00128.x. 8301602

[odi70063-bib-0006] Hirshberg, A. , A. Shnaiderman‐Shapiro , I. Kaplan , and R. Berger . 2008. “Metastatic Tumours to the Oral Cavity ‐ Pathogenesis and Analysis of 673 Cases.” Oral Oncology 44, no. 8: 743–752. 10.1016/j.oraloncology.2007.09.012. 18061527

[odi70063-bib-0007] Lombardi, N. , A. Sardella , and G. Lodi . 2021. “Tongue Deviation and Dysarthria in Painless Patient.” European Annals of Otorhinolaryngology, Head and Neck Diseases 139: S18797296(21)002258. 10.1016/j.anorl.2021.07.013. 34625390

